# Methods for implementing integrated step-selection functions with incomplete data

**DOI:** 10.1186/s40462-024-00476-8

**Published:** 2024-05-09

**Authors:** David D. Hofmann, Gabriele Cozzi, John Fieberg

**Affiliations:** 1 Department of Evolutionary Biology and Environmental Studies, University of Zurich, Winterthurerstrasse 190, 8057 Zurich, Switzerland; 2 Botswana Predator Conservation Program, Wild Entrust, Private Bag 13, Maun, Botswana; 3 Department of Fisheries, Wildlife, and Conservation Biology, University of Minnesota, St. Paul, MN USA

**Keywords:** Animal movement, GPS data, Imputation, Incomplete data, Missing fixes, Step-selection analyses, Step-selection functions

## Abstract

Integrated step-selection analyses (iSSAs) are versatile and powerful frameworks for studying habitat and movement preferences of tracked animals. iSSAs utilize integrated step-selection functions (iSSFs) to model movements in discrete time, and thus, require animal location data that are regularly spaced in time. However, many real-world datasets are incomplete due to tracking devices failing to locate an individual at one or more scheduled times, leading to slight irregularities in the duration between consecutive animal locations. To address this issue, researchers typically only consider bursts of regular data (i.e., sequences of locations that are equally spaced in time), thereby reducing the number of observations used to model movement and habitat selection. We reassess this practice and explore four alternative approaches that account for temporal irregularity resulting from missing data. Using a simulation study, we compare these alternatives to a baseline approach where temporal irregularity is ignored and demonstrate the potential improvements in model performance that can be gained by leveraging these additional data. We also showcase these benefits using a case study on a spotted hyena (*Crocuta crocuta*).

**Supplementary Information:**

The online version contains supplementary material available at 10.1186/s40462-024-00476-8.

## Introduction

Understanding how animals move across the landscape, what habitats they prefer, and what resources they select are fundamental questions in movement ecology [56]. Thanks to recent advances in animal tracking [5, 11, 76] and remote sensing technologies [64, 72], new opportunities and analytical tools have emerged for studying how animals move and interact with their environment [41, 57, 71]. Methods commonly used to analyze animal movement data, including step-selection analyses [27, 28, 70] and hidden Markov models [53], require **animal locations** (terms in bold at first occurrence are defined in Table 1) that are collected at a constant sampling frequency, leading to data that are equally spaced in time. Yet, it is common to encounter missing locations in most telemetry data sets [29, 33, 73], which introduces unwanted irregularities in the duration between successive locations. Thus, there is a need for analytical tools that enable the analysis of such data, while mitigating potential biases arising from temporal irregularity introduced through missing animal locations.

Step-selection analyses (SSAs) are widely used to study animals’ movement capacities and habitat-selection patterns [28, 70]. Straight-line segments connecting consecutive animal locations, referred to as **steps**, form the basic building blocks of the statistical likelihood in SSAs. Specifically, SSAs model the probability *u* of finding an individual at location *s* at time $t+1$, given the animal’s past positions at time *t* and $t-1$, $s_t$ and $s_{t-1}$, respectively:

1 $$\begin{aligned} u(s_{t+1}) = \frac{\phi (s_{t+1}, s_t, s_{t-1}; \gamma )w(x(s_{t+1}); \beta )}{\int _{s \in G}\phi (s_{t+1}, s_{t}, s_{t-1}; \gamma )w(x(s_{t+1}); \beta )ds} \end{aligned}$$

Here, the function $\phi$ represents an animal’s **movement kernel** which is usually expressed in terms of **step-length** and **turning-angle** distributions, with $\gamma$ representing parameters in these distributions. The function *w* is the **habitat-selection function** and reflects an animal’s preferences $\beta$ for environmental characteristics *x* at location $s_{t+1}$. In most applications, *w* is modeled as a log-linear function of *x*, taking the form $w = exp(x^\top \beta )$. The integral in the denominator of Eq. 1 ensures that *u* is a proper probability distribution (i.e., that it integrates to 1). Following Michelot [52], we call the product $\phi \times w$ the step-selection function (SSF), as it highlights that the probability of finding an animal at a certain location depends on both the animal’s movement kernel and its habitat-selection function.

Given a series of **observed steps**, finding the movement and habitat-selection parameters that maximize the likelihood in Eq. 1 requires approximating the integral in the denominator for each observed step. A variety of numerical integration techniques can be used for this purpose [52], but a common approach is to combine observed steps with **random steps** generated by sampling step lengths and turning angles from parametric distributions informed by the data [28, 70]. Environmental conditions at observed steps are then contrasted with environmental conditions at random steps in a (mixed effects) conditional logistic regression framework [28, 54]. To jointly estimate parameters in $\phi$ and *w*, movement descriptors (e.g., step length (sl), its natural logarithm (log(sl)), and the cosine of the turning angle (cos(ta))) can be included in the conditional logistic regression model, and their estimated coefficients can be used to update the initial (tentative) step-length and turning-angle distributions [3, 22, 27]. The specific descriptors that need to be included depend on the assumed step-length and turning-angle distributions [for more details, see Appendix C of 27]. This approach to estimating parameters of the SSF, termed *integrated* SSA (or iSSA) by Avgar [3], is similar to using importance sampling to approximate the integral in 1 [52] and is readily accessible through the R-package amt [67].

SSAs have proven extremely effective in numerous ecological studies [70], providing insights into seasonal space use [25, 73], resource selection during distinct behavioral phases [1, 7, 16, 24], and the effects of landscape familiarity or memory on animal movements [43]. A model parametrized using iSSA resembles a fully mechanistic movement model that can be used to simulate space use under novel conditions [3, 36, 65, 66]. This characteristic has made iSSAs a useful tool for quantifying landscape resistance and identifying movement corridors [10, 34, 36, 80].

**Table 1:** Glossary of terms

Term	Definition
Animal locations	A series of telemetry data points that include date, time, longitude, and latitude information, describing when and where an animal was observed or recorded.
Step	A straight line connecting two consecutive locations.
Observed step	A step that connects two observed animal locations.
Random step	A step that connects an observed animal location with a random location. Random locations are typically generated by combining an observed animal location with random step lengths and turning angles.
Step length	The Euclidean distance of a step.
Turning angle	A measure of the change in direction between two consecutive steps.
Habitat-selection function	A probabilistic description of an animal’s habitat preferences. It describes how an animal selects habitat when not constrained by its movement capacity. Also referred to as movement-free habitat-selection function.
Movement kernel	A probabilistic description of an animal’s movement capacity. It describes how an animal would move when not constrained by habitat selection. Also referred to as selection-free movement kernel.
Trajectory	A sequence of animal locations collected on the same individual.
Step duration	The time interval associated with a particular step, i.e., the time elapsed between two consecutive animal locations.
Regular animal locations	A series of animal locations that have been obtained at regularly spaced time intervals, such as every hour.
Irregular animal locations	A series of animal locations collected at irregular time intervals.
Regular step durations	Step durations that occur when animal locations are successfully collected at regular time intervals.
Irregular step durations	Step durations that occur when animal locations are not successfully collected at regular time intervals.
Missingness	The fraction of animal locations that should have been collected but, for some reason, were not. For example, if only eight out of ten expected animal locations were successfully collected, the missingness would be 0.2 (i.e., 20%).
Forgiveness	The maximum step duration, measured in multiples of the regular step duration, a modeler is willing to include in the step-selection analysis. A modeler with a forgiveness of one, for instance, only considers regular steps, while a modeler with a forgiveness of two would consider irregular steps up to twice the regular step duration.
Burst	A sequence of consecutive animal locations equally spaced in time and with steps where the step duration does not exceed the forgiveness.
Valid step	A step for which a step length, turning angle, and step duration can be computed, and for which the step duration does not exceed the forgiveness of the modeler. These steps can be used for step-selection analysis.

Terms in the glossary are printed in bold at first occurrence in the main text. Definitions are always given in the context of step selection functions (SSFs)

A key assumption when conducting an iSSA is that the data have been collected at a constant sampling frequency, thus producing **trajectories** with **regular step durations** ($\Delta t$; [28, 70]). Here, we refer to such data as **regular animal locations**, and without loss of generality, we assume the regular step duration to be one (i.e., $\Delta t = 1$). Regular step durations ensure that step lengths and turning angles are independent of the step duration, and therefore, steps can be pooled when estimating movement parameters. Since animal locations are usually obtained using automated tracking devices, such as GPS collars programmed to record data at regular intervals, satisfying this assumption seems straightforward. In reality, however, device limitations often imply that some of the aspired datapoints fail to be collected, thus introducing **missingness** and confronting researchers with **irregular animal locations** and **irregular step durations** [29]. In a comprehensive study, Hofman [33] showed that across 3000 GPS devices and 160 species, the average success rate of obtaining a scheduled animal location was 78% (implying a missingess of 22%), thus highlighting that irregular animal locations are a frequent phenomenon in ecological studies.

**Fig. 1:**
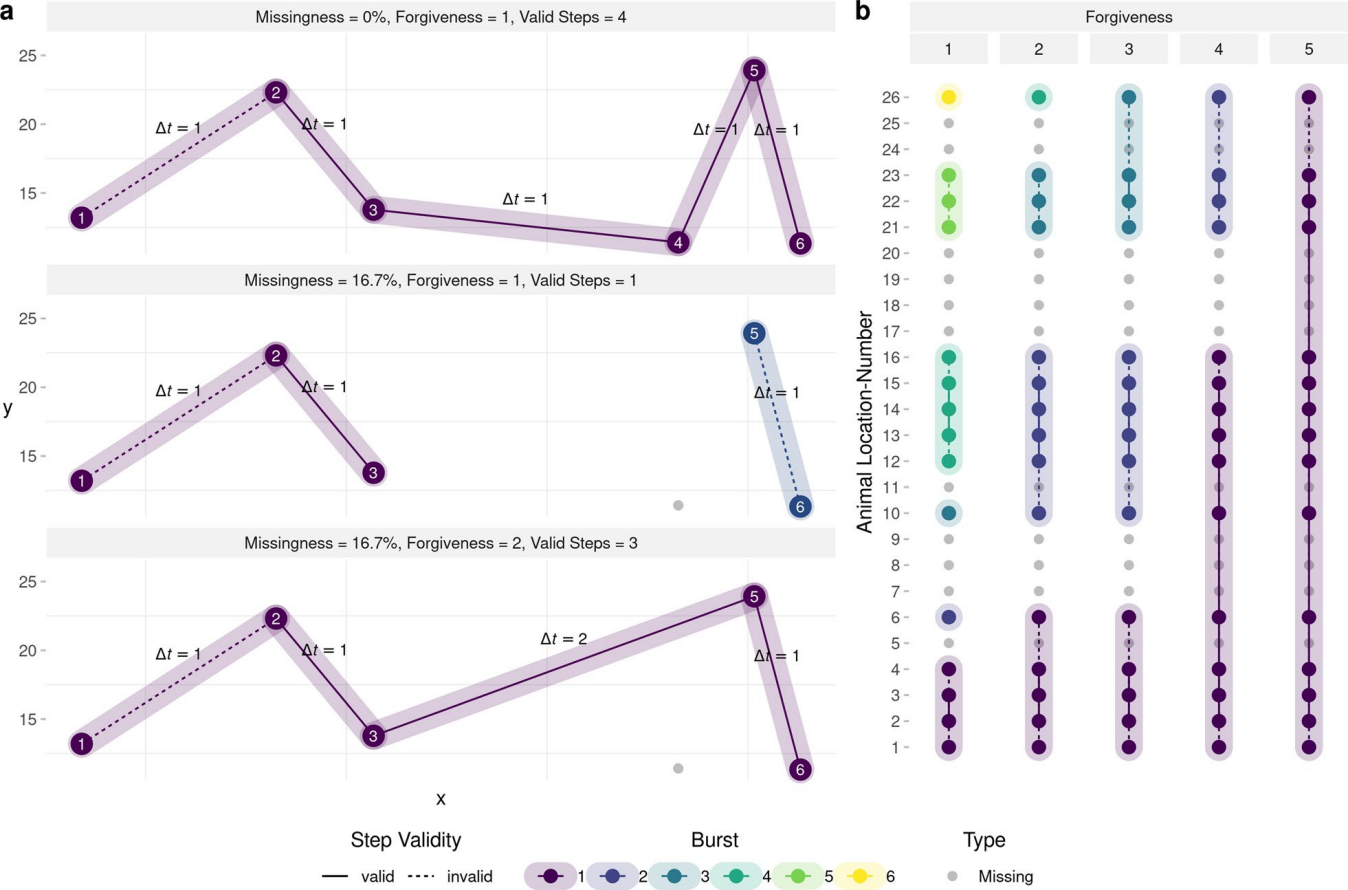
**a** Demonstration of how missingness affects the number of valid steps that can be used for step-selection analyses under different levels of forgiveness. The upper panel depicts a trajectory with zero missing locations. That is, all aspired locations were successfully collected on a regular interval (yielding a regular step duration of $\Delta t = 1$). This trajectory produces four valid steps that can be included in the iSSF model and one invalid step that has to be omitted because it has no turning angle associated with it. In the central panel, animal location 4 was not obtained, introducing a missingness of 16.7%. If the modeler has a forgiveness of one, only a single step can be included for further analysis, as all other steps are invalid (either because no turning angle can be computed or because step durations exceed the forgiveness). If, however, the modeler exhibited a forgiveness of two, such as in the lower panel, a total of three steps could be obtained for further analysis. **b** Conceptual illustration of how increasing the forgiveness allows one to retain additional steps that can be used for step-selection analysis. The sequence of dots resembles the sequence of locations that were scheduled to be collected (e.g. using a GPS device), with the lines representing hypothetical steps. Because not all locations were successfully obtained (gray dots), there is missingness. Depending on the forgiveness level, already a single missing location enforces the introduction of a new burst, which leads to the loss of several steps. In addition, some of the remaining steps are invalid (dotted) because they are lacking a turning angle. By increasing the forgiveness, a modeler is willing to retain steps that exceed the regular step duration by a certain threshold, which enables them to obtain longer bursts and increase the number of steps that can be used for further analysis. In the figure, forgiveness increases from left to right

**Fig. 2:**
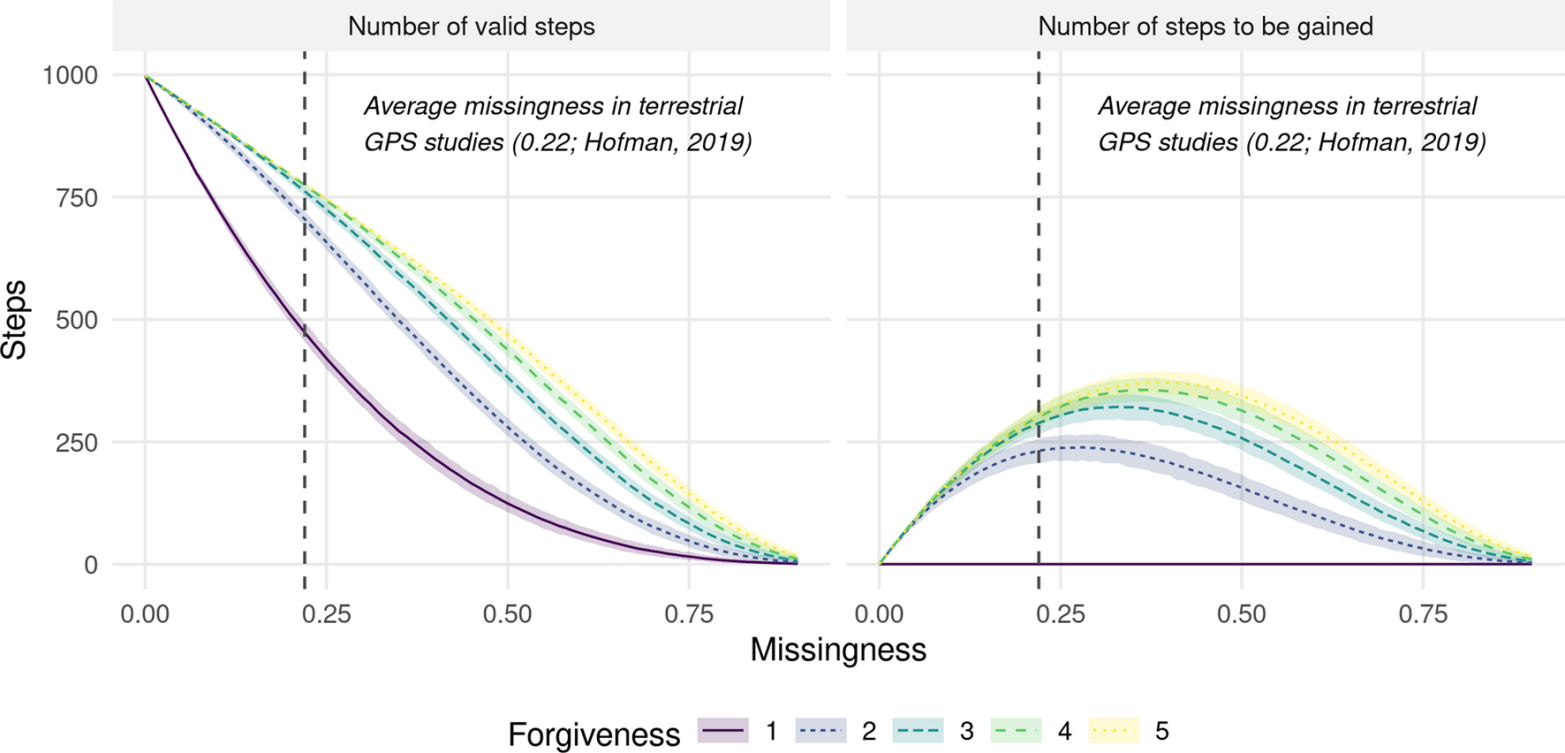
Illustration of how missingness in animal location data reduces the number of valid steps that can be used in step-selection analyses (left panel) and how increasing forgiveness helps to retain additional steps that are otherwise omitted (right panel). At a missingness of 0, 998 valid steps can be obtained from the total of 1000 animal locations. At higher missingness, step durations become irregular, which means that the number of valid steps decreases substantially. However, if the modeler is willing to increase their forgiveness, additional steps can be gained. The right panel shows the number of valid steps that is gained when increasing the forgiveness from 1 to 2, 3, 4, and 5, respectively. Ribbons indicate the 95%-percentile intervals derived from 1000 replicates

It is generally recommended that, in the case of such irregular data, researchers should only retain **bursts** of steps with regular step durations (possibly with some tolerance) and discard the rest [70]. We will refer to this modeling approach as having a **forgiveness** level of one, indicating that only steps with step durations $\Delta t = 1$ are retained for further analysis. In R, the amt package provides the function track_resample specifically for identifying bursts of steps with regular step durations [67]. The main drawback of this approach is that it may result in a substantial amount of data being discarded (Fig. 1 and Fig. 2). For instance, consider a hypothetical trajectory in which location 4 is missing (Fig. 1a). The absence of this location prevents the computation of a step between locations 3 and 4, as well as between locations 4 and 5. Furthermore, without these steps, it becomes impossible to compute a turning angle for the step between locations 5 and 6. Consequently, the lack of a single location reduces the effective sample size, which is the number of **valid steps**, by three. Assuming that animal locations are missing at random, a missingness level of 25% causes the number of valid steps to drop by $58\%$ (Fig. 2). A modeler willing to increase their level of forgiveness to two (i.e., allowing for inclusion of steps with $\Delta t \le 2$) would be able to increase the number of valid steps by $57\%$ (Fig. 1 and Fig. 2), therefore achieving a substantial gain in effective sample size. The ability to capitalize on irregular data is likely to be particularly important for applications where data are already limited, such as, for instance, when modeling dispersing individuals [13, 26, 63]. However, increasing the forgiveness also implies that step durations of the retained steps become irregular, thus necessitating appropriate tools to account for such irregularity.

Various methods have been employed in the past to address temporal irregularities in animal location data. These may serve as valuable starting points for developing approaches that enable the integration of irregular data with iSSFs.

1. *Imputation*: An intuitive solution is provided by McClintock [50], who suggests fitting a continuous-time correlated random walk movement model [38] to the collected data and to use the fitted model to impute missing fixes. By imputing missing locations, the analysed trajectories become entirely regular again and can be analysed using traditional techniques. This approach, which we coin the *imputation* approach, is readily available through the R-package crawl [37], yet has only been tested for use with hidden Markov movement models and not with iSSFs [50].
2. *Naïve*: Another approach is outlined by Munden [55], who introduced time-varying iSSFs. In this framework, a change-point detection algorithm is applied to the series of observed animal locations to identify distinct decision points where the animal turns [55, 61]. Steps are then created to represent straight-line movements in-between these decision points, but because decision points are not regularly spaced in time, the resulting step durations are irregular. Thus, step durations are treated as random variables, and, instead of generating random steps by sampling step lengths and turning angles, the authors generate random steps by sampling step durations, step speeds, and turning angles. The underlying assumption is that step lengths scale linearly with step duration, and can therefore be meaningfully represented by combinations of step speeds and step durations. Although this approach was developed with ultra-high-frequency data in mind, we might *naïvely* apply it more broadly to the case of missing data if we believe the presumed linear relationship to hold true (this assumption may be reasonable when step durations are short, but it is unlikely to hold for longer step durations since an animal’s path will deviate from a straight line between successive locations). Hence, we propose, with our *naïve* approach, to scale the generated random steps by the observed step duration.
3. *Dynamic+Model*: Instead of generating random steps by sampling step lengths and turning angles from distributions fitted to a single step duration, one may choose to fit separate distributions to steps of different durations, thus acknowledging potentially non-linear relationships between step duration and step lengths or turning angles. Because random steps in this approach are sampled using different *tentative* distributions, it is necessary to include interactions between step durations and other step descriptors (e.g., sl, log(sl), and cos(ta)) in the conditional logistic regression model to allow updating the distributions to the different step durations. We therefore refer to this approach as the *dynamic+model* approach, highlighting that step-length and turning-angle distributions are dynamically adjusted to observed step durations and that the step duration is included as a modifier of the coefficients of step descriptors in the regression model.
4. *Multistep*: Finally, we propose a *multistep* approach, where random steps of varying step durations are generated by stitching together sequences of random steps from the regular step duration. For example, one can generate a random step of duration $\Delta t = 2$ by combining two random steps of step duration $\Delta t = 1$.

Our goal with this article is to reassess the practice of discarding irregular animal locations in iSSFs and to investigate whether retaining irregular data could, in fact, serve to improve model performance. Our hypothesis is that even irregular data contains valuable information on habitat and movement preferences that could be leveraged if appropriate methods are applied. To test this notion, we conducted a comprehensive simulation study where we simulated regular animal location data with known movement and habitat parameters. We then introduced varying levels of missingness and applied iSSFs to estimate simulation parameters. Specifically, we employed the four alternative iSSF approaches outlined above and compared them to the traditional approach of including only bursts of regular data and to an uncorrected approach that simply ignored irregular step durations when using a forgiveness level > 1. To examine the impact of different landscape configurations on derived estimates, we ran our simulations for different levels of spatial autocorrelation. The use of simulations instead of real data had the benefit that underlying parameters of the movement kernel and habitat-selection function were known, which allowed us to assess the reliability of different methods in retrieving true simulation parameters under different conditions (sensu [42]). We then compare the traditional approach (using only bursts of steps with regular step durations) to the best-performing approach (with irregular data) using GPS locations collected on a spotted hyena (*Crocuta crocuta*).

We anticipated that increasing forgiveness without adjusting for the introduced irregularity would entail a bias-variance trade-off. Specifically, we anticipated that increasing forgiveness would allow improving estimator precision, but at the cost of introducing bias due to failing to account for irregular sampling intervals. We expected this bias to be particularly pronounced at high levels of missingness. Furthermore, we hypothesized that accounting for irregularity in the *naïve*, *dynamic+model*, and *multistep* approaches would improve model accuracy, while alleviating potential bias, thus providing an effective means of incorporating additional data. Because the *imputation* approach relied on an intermediate movement model to predict missing animal locations, we had no prior expectations for how well it would perform.

## Methods

**Fig. 3:**
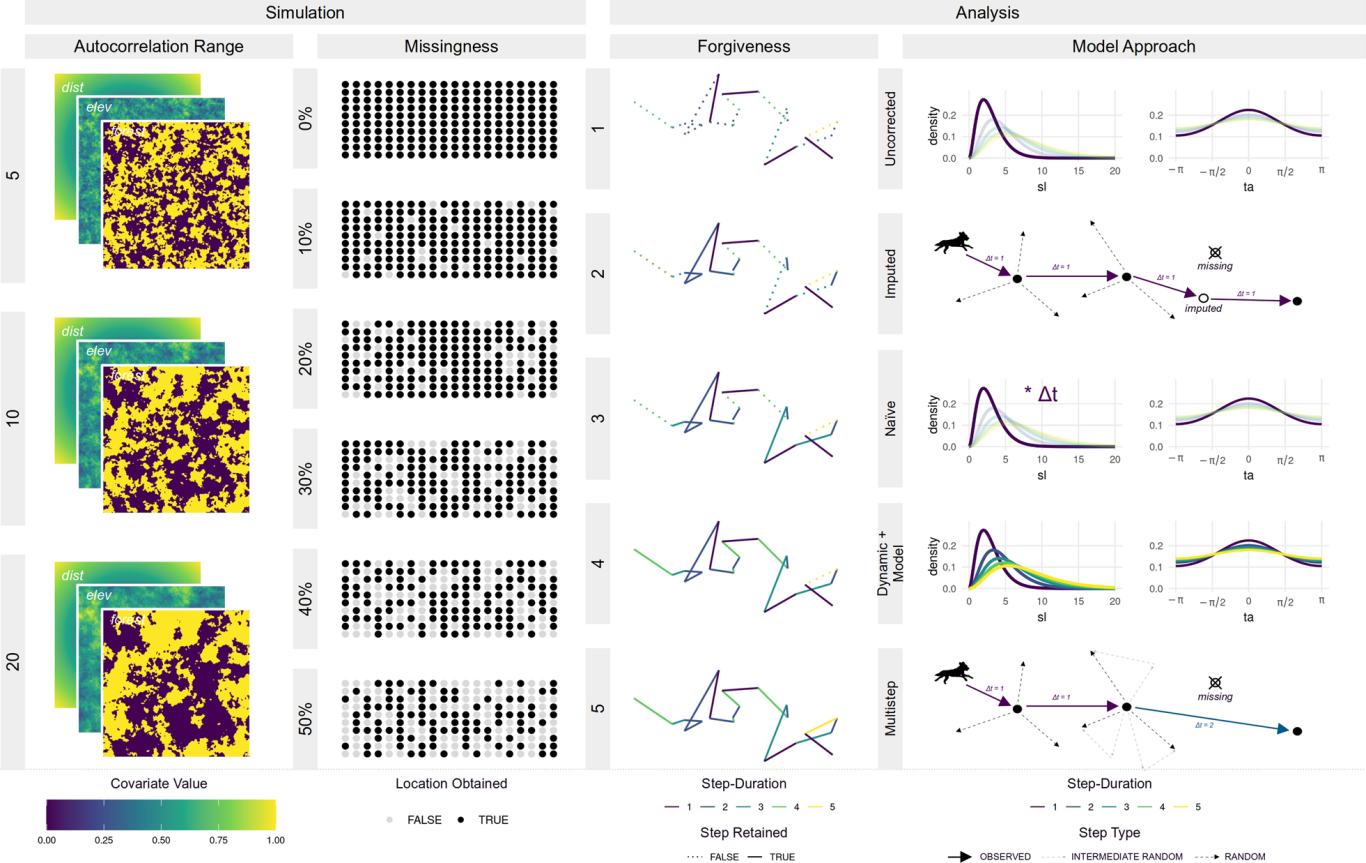
Illustration of the study design. We varied the autocorrelation range when simulating spatial covariates from 5 to 20 and tested for different missingness scenarios (ranging from 0% to 50% missing locations). To investigate how increasing forgiveness (i.e., the willingness to include steps with duration above the regular step duration) influenced model results, we varied its value from 1 (regular step selection) to 5 (considering steps that are five times the regular step duration). Finally, we tested five different methods to account for potential biases introduced by including irregular steps. This gave 3 x 6 x 5 x 5 = 450 combinations, each of which we replicated 100 times. We assumed step lengths (sl) to follow a gamma distribution, whereas turning angles (ta) followed a von Mises distribution

We implemented the simulation study in the programming language R version 4.3.2 [62] and achieved parallelization of simulation-runs using the R-package pbmcapply [46]. We generated figures using the ggplot2 [74], ggpubr [40], and ggh4x [6] R-packages. We manipulated raster data and computed spatial distances using the R-package raster [32]. An overview of the simulation design and the different iSSF approaches is presented in Fig. 3 and all codes to reproduce this study are available through an online repository [35].

### Landscape simulation

**Fig. 4:**
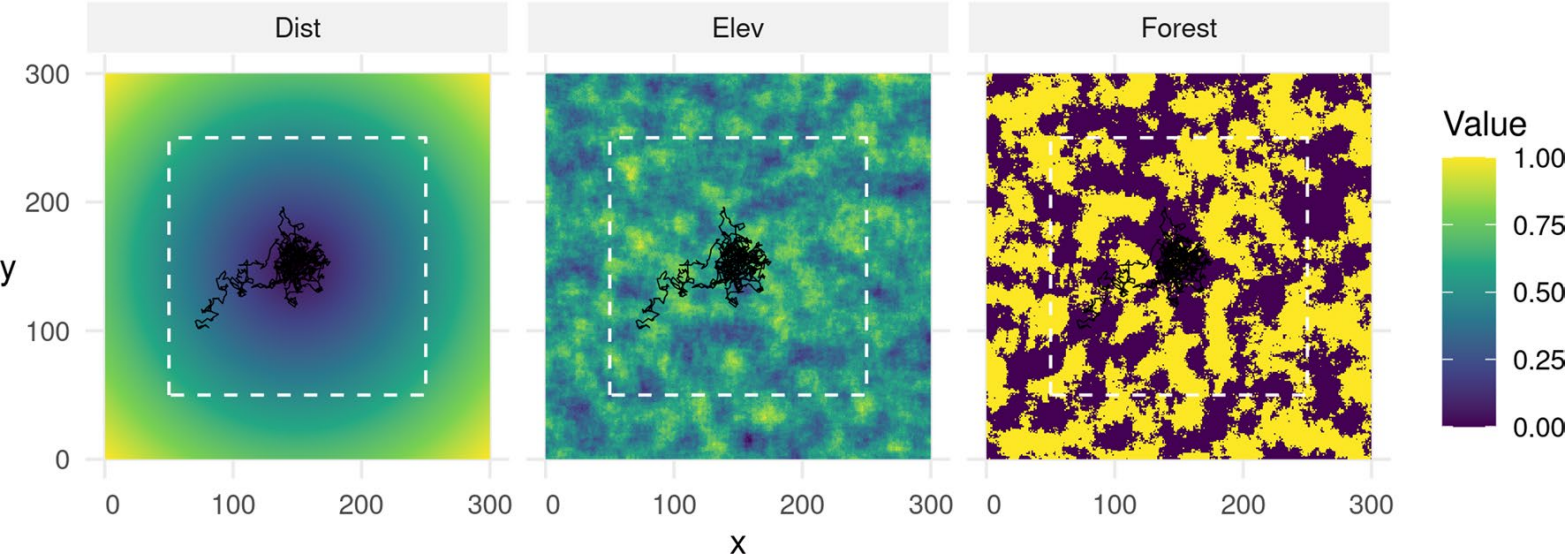
Example of a landscape configuration across which we simulated movement trajectories. All simulated layers had a resolution of 300 x 300 pixels. The distance layer indicated the distance to the center of the landscape and served to simulate attraction. The elevation and forest layers were simulated by sampling pixel-values from a normal distribution and applying a moving window to achieve spatial autocorrelation. Simulated individuals were initiated within the white dashed rectangle, which ensured that they would not be released directly at a map border. Simulated individuals were attracted to the landscape’s center, preferred elevated areas, and avoided areas covered by forest. The black line shows the simulated trajectory associated with the visualized landscape configuration (cfr. Section Movement simulation)

We simulated a virtual landscape comprising two continuous and one categorical (binary) spatial layers, each with a resolution of 300 $\times$ 300 pixels (Fig. 4) and spanning across x- and y-coordinates from 0 to 300. The first layer, Dist (continuous), quantified the distance to the center of the virtual landscape ($x = 150$, $y = 150$), and can be understood as a point of attraction, such as, for instance, the center of an animal’s home-range. The second layer, Elev (continuous), resembled an elevation layer and was simulated by sampling random pixel-values from a normal distribution. To achieve spatial autocorrelation, we applied a circular moving window with radius *r* within which we tallied pixel-values. We varied *r* from 5, to 10, to 20, depending on the simulated level of autocorrelation (Appendix 1: Figure S1). The third layer, Forest (categorical), represented areas covered by woodland and was simulated similarly to the Elev layer, but we binarized the layer by setting all simulated values above the 50% quantile to forest and all other values to non-forest (our reference class). We normalized values of all simulated layers to a range between zero and one and replicated the simulation of each layer 100 times per autocorrelation scenario, thus producing 300 unique landscape configurations.

### Movement simulation

To simulate movement across the virtual landscape, we employed the iSSF simulation algorithm developed by Signer [66] and applied in Hofmann [36]. This procedure consists of a sequence of five steps that are repeated *n* times to generate a movement trajectory. In step one, we generated a random starting location by sampling random x- and y-coordinates on the simulated landscape. To prevent starting points near map borders, we restricted sampled locations to x- and y-coordinates between 50 and 250 (white dotted rectangle in Fig. 4). In step two, we generated a set of 1000 random steps originating from the current location, by sampling turning angles from a von Mises distribution with concentration parameter $\kappa = 0.5$ and step lengths from a gamma distribution with shape parameter $k = 3$ and scale parameter $\theta = 1$. In step three, we extracted covariate values at the end of each random step from the underlying covariate layers. In step four, we assigned to each step *j* a probability $\pi _j$ of being selected using the equation below:

2 $$\begin{aligned} \pi _j = \frac{exp(\beta ^\top x_j)}{\sum _{i = 1}^J exp(\beta ^\top x_i)} \end{aligned}$$

Here, $\beta$ represents the vector of habitat-selection parameters and $x_j$ the covariate value at the end of the *j*^th^ step. The probability of a step being selected thus depended on its associated covariate values, the covariate values of all other random steps, and the simulated preferences $\beta$. We defined the habitat-selection parameters as $\beta _{dist} = -20$, $\beta _{elev} = 0.5$, and $\beta _{forest} = -1$. That is, simulated individuals were attracted to the landscape’s center, preferred elevated areas, and avoided areas covered by forest. In step five, we sampled one of the random steps based on predicted probabilities and computed the simulated individual’s new position. We then repeated steps two through five until the trajectory comprised a total of 1000 steps. Each simulated step was assumed to have a step duration of exactly $\Delta t = 1$. We repeated the simulation for each of the 300 simulated landscapes, producing 300 unique trajectories (example trajectory presented in Fig. 4).

### Data rarefication

To simulate missingness, we rarefied the trajectories by randomly removing a fixed fraction of animal locations. To assess the impact of different degrees of missingness, we varied the fraction of removed data from 0% (complete dataset) to 50% in increments of 10%. The random removal of animal locations introduced temporal irregularity, such that the resulting step durations differed depending on the time elapsed between remaining fixes. We replicated the rarefication of each trajectory 100 times.

### Computing bursts

We used the rarefied data to compute bursts consisting of a sequence of animal locations with step durations that did not exceed the forgiveness value. To test how different levels of forgiveness impacted our results, we varied forgiveness from 1 (maximum allowed step duration was $\Delta t = 1$) to 5 (maximum allowed step duration was $\Delta t = 5$). As an example, if the forgiveness was 1, any step with step duration $\Delta t > 1$ resulted in a new burst. If the forgiveness was 2, in contrast, step durations of up to $\Delta t = 2$ were allowed before a new burst was introduced (Fig. 1b). Within each burst, we calculated step lengths and turning angles. However, due to the grouping of steps into bursts, the orientation of the first step within each burst relative to the previous step could not be determined. As a result, this step always lacked a turning angle and was considered invalid (Fig. 1b).

### Fitting distributions

Based on the steps retained within bursts, we parametrized tentative step-length and turning-angle distributions. Specifically, we used the fit_distr function from the amt package [67], which is a wrapper function for the fitdist function from the fitdistrplus package [19], and fitted a gamma distribution to step lengths and a von Mises distribution to turning angles. Notably, we employed two different fitting procedures:

1. *Regular Distributions:* In this procedure, we fitted parametric distributions considering only step lengths and turning angles from steps that exhibited a step duration of $\Delta t = 1$ (i.e., the regular step duration). Any steps with irregular step durations ($\Delta t > 1$) were discarded and did not affect distributional parameter estimates. This represents the traditional procedure in iSSFs where only regular bursts of animal locations are considered when estimating tentative movement parameters.
2. *Dynamic Distributions:* In this procedure, we fitted separate parametric distributions to step lengths and turning angles from steps of different step durations. That is, we parametrized separate turning-angle and step-length distributions representative of steps with durations of $\Delta t = 1, 2, 3, 4$ and 5 (which corresponds to the maximum forgiveness level we tested for). Some step durations only rarely occurred at low levels of missingness, thus complicating parametrization of the associated distributions. To facilitate estimation of dynamic distribution parameters across all $\Delta t$ (Appendix 1: Figure S2), we resampled data to different step durations using the track_resample function from the amt package [67] before fitting tentative parameters. This ensured a sufficient number of steps for each step duration to estimate associated parameters. An alternative approach would be to increase missingness in the data even further, thus introducing a larger number of longer step durations.

### Step-selection functions

We implemented a baseline *uncorrected* approach and four alternative iSSF approaches that mainly differed in the way in which random steps were generated, but sometimes also in the model call that was used to estimate parameters (Fig. 3). In the *uncorrected* approach, we treated data as if they were regular, ignoring potential issues arising from having variable step durations. When forgiveness was one, this approach corresponded to the traditional iSSF approach. All other approaches were targeted towards reducing potential biases arising from the inclusion of steps with irregular step durations. Irrespective of the approach employed, we paired each observed step with a total of 200 random steps:

- *Uncorrected*: In the uncorrected approach, we generated random steps by sampling step lengths and turning angles from statistical distributions fitted to steps with step durations of $\Delta t = 1$, regardless of the forgiveness value or observed step durations. Thus, this approach ignored any potential effect of step duration when generating random step lengths and turning angles.
- *Imputed*: In this approach, we sampled step lengths and turning angles from statistical distributions fitted to observed steps with a step duration of $\Delta t = 1$. However, prior to generating random steps, we imputed missing fixes using predictions from a simple movement model. Specifically, we fitted a single-state movement model [38] to the simulated trajectories and used the parametrized model to predict coordinates for all missing animal locations. For this, we used the functions crwFit and crwPredict from the crawl R-package [37]. Although the crwFit function provides capabilities to incorporate location measurement error, we assumed animal locations were measured without error. The imputation resulted in a complete dataset without any missing animal locations, such that each trajectory consisted of a single continuous burst of locations equally spaced in time. As such, the imputation approach is not affected by the forgiveness level.
- *Naïve*: In the *naïve* approach, we again sampled step lengths and turning angles from regular distributions fitted to steps with step durations of $\Delta t = 1$. However, we linearly scaled the sampled step lengths depending on the step durations of the observed steps. For instance, we doubled the sampled step lengths for any observed step with a step duration of $\Delta t = 2$. This approach naïvely assumed that step lengths scale linearly with step durations, which is unlikely to be true, as most animals don’t move in straight lines between successive observations. Furthermore, the linear approximation is likely to get worse as step duration increases (i.e., as the forgiveness value increases). Since it is not clear how turning angles should scale with step duration, we did not adjust the sampled turning angles.
- *Dynamic+Model*: In the *dynamic+model* approach, we sampled step lengths and turning angles from dynamic distributions that were fit to different step durations. That is, for observed steps with step duration of $\Delta t = 2$, we sampled step lengths and turning angles from distributions fit to observed steps with $\Delta t = 2$. We then included interactions between the step duration and other step descriptors (e.g., sl, log(sl), cos(ta)), allowing us to update movement parameters for each step duration separately. To avoid numerical instabilities with the conditional logistic regression model, we only included steps with durations $\Delta t > 1$ if the respective duration was represented at least 5 times in the rarefied dataset.
- *Multistep*: In the *multistep* approach, we sampled step lengths and turning angles from statistical distributions fitted to observed steps with step durations of $\Delta t = 1$. We then generated a sequence of random steps such that their combined step duration equaled the step duration of each observed step. For instance, for an observed step with step duration of $\Delta t = 2$, we generated sets of two random steps, which we then concatenated into a “random path”. The paths were then simplified to straight lines connecting the first and last coordinate of each path, which represented the final random step.

Together, an observed step and its 200 associated random steps formed a *stratum* that received a unique ID. At the end of each step, we extracted covariate values from the underlying covariate layers.

### Conditional logistic regression model

We estimated movement and habitat-selection parameters for the simulation scenarios presented in Fig. 3 using conditional logistic regression, implemented using the clogit function in the R-package survival [69]. We defined a binary response variable (observed) indicating if a step was an observed (scored 1) or a random step (scored 0) and used the step’s ID as a stratification variable. We included habitat covariates (dist, elev, forest) and step descriptors (sl, log(sl), cos(ta)) as predictors in the regression model. For the *dynamic+model* approach, we also included interactions between the step duration, coded as a factor, and step descriptors. To update tentative movement parameters (denoted by the subscript _0_) and obtain the selection-free movement kernel (denoted by the ^ symbol), we employed the formulas provided in [3, 27]. Specifically, we updated the shape ($\hat{k}$) and scale ($\hat{\theta }$) parameters of the step-length distribution (gamma) using:

$$\begin{aligned} \hat{k}= & {} k_0 + \beta _{log(sl)} \\ \hat{\theta }= & {} \frac{1}{\frac{1}{\theta _0} - \beta _{sl}} \end{aligned}$$

We updated the concentration parameter ($\hat{\kappa }$) of the turning-angle distribution (von Mises) using:

$$\begin{aligned} \hat{\kappa } = \kappa _0 + \beta _{cos(ta)} \end{aligned}$$

We kept track of the estimates of the updated movement ($\hat{k}$, $\hat{\theta }$, and $\hat{\kappa }$) parameters and the habitat-selection ($\hat{\beta }_{dist}$, $\hat{\beta }_{elev}$, $\hat{\beta }_{forest}$) parameters, and compared them to the true simulation parameters. We also quantified model accuracy via the root-mean-square error (RMSE).

## Results

**Fig. 5:**
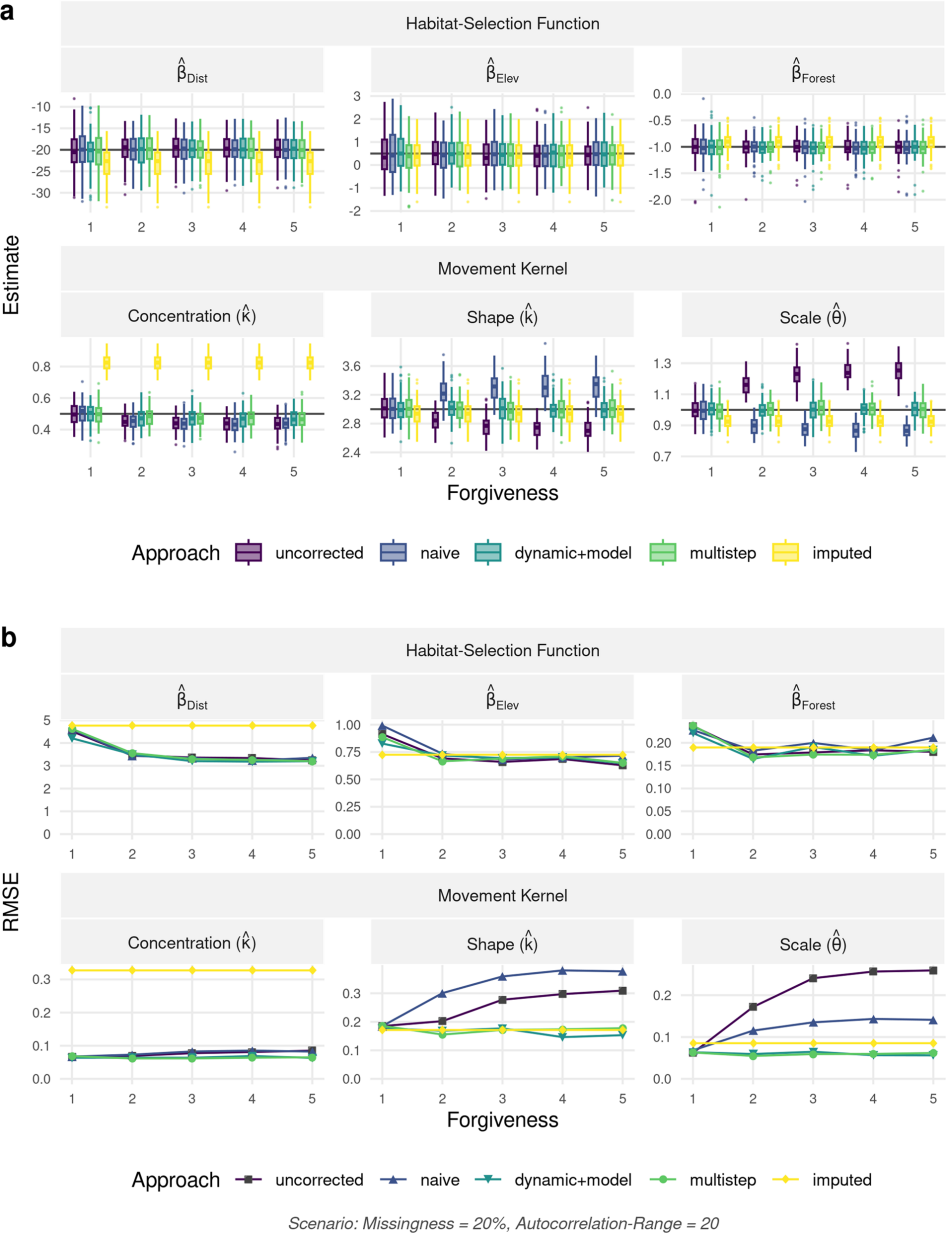
**a** Parameter estimates and **b** root mean-square error (RMSE) with regard to the movement kernel and habitat-selection function as a function of forgiveness. Results are shown for the scenario with landscape autocorrelation of 20 and missingness of 20%. The movement kernel comprised of a gamma distribution with shape parameter *k* and scale parameter $\theta$ governing the step-length distribution and a von Mises distribution with concentration parameter $\kappa$ governing the turning-angle distribution. Habitat-selection was based on three covariates, namely a Distance, Elevation, and a Forest layer. Estimates are shown for the five different approaches we tested for. The uncorrected approach ignored the fact that higher forgiveness implied temporal irregularity in the data, while all other approaches attempted to correct for the potential biases introduced by temporal irregularity. Note, the imputation approach is not affected by the forgiveness level, since it always starts with a full trajectory

**Fig. 6:**
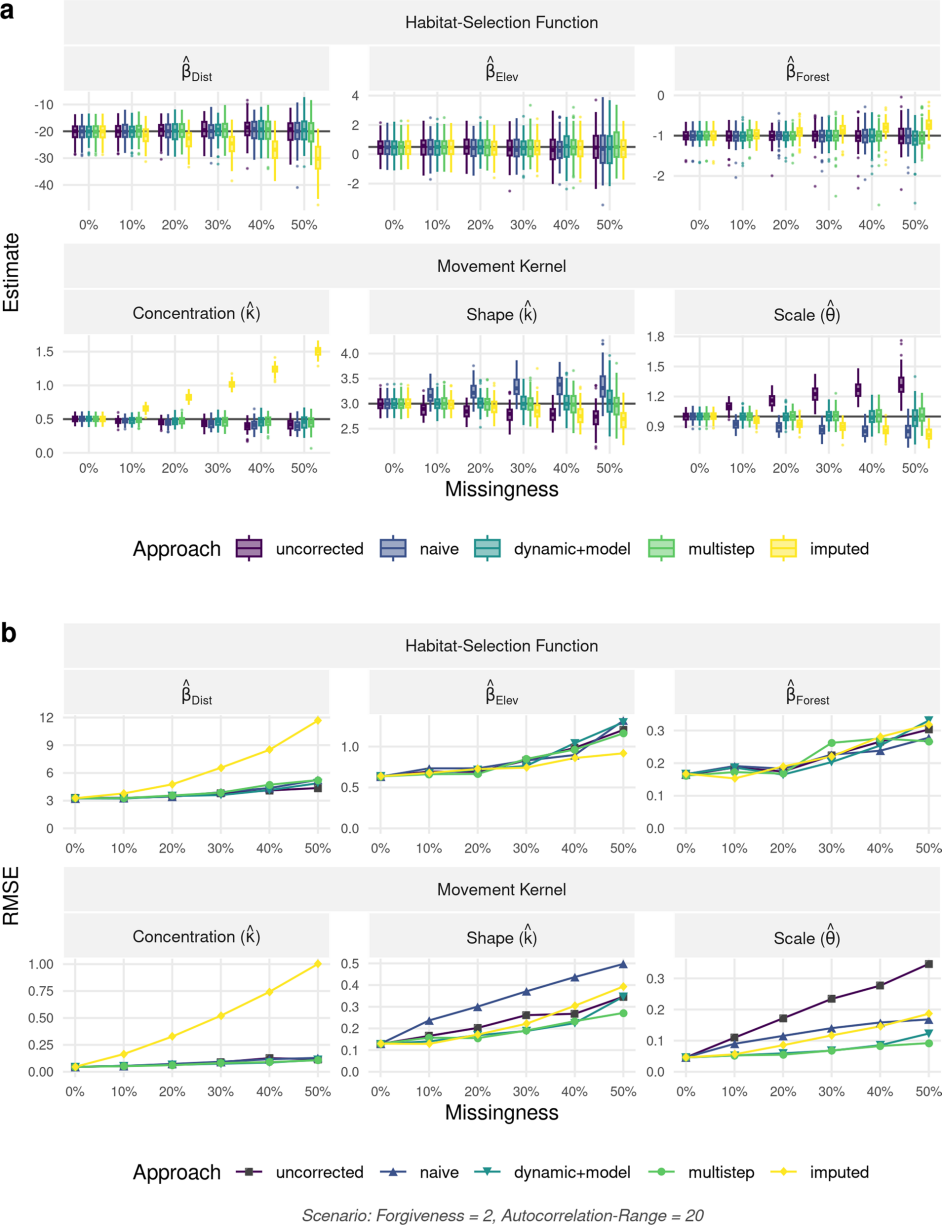
**a** Parameter estimates and **b** root mean-square error (RMSE) with regard to the movement kernel and habitat-selection function as a function of missingness. Results are shown for the scenario with landscape autocorrelation of 20 and forgiveness of 2. The movement kernel comprised of a gamma distribution with shape parameter *k* and scale parameter $\theta$ governing the step-length distribution and a von Mises distribution with concentration parameter $\kappa$ governing the turning-angle distribution. Habitat-selection was based on three covariates, namely a Distance, Elevation, and a Forest layer. Estimates are shown for the five different approaches we tested for. The uncorrected approach ignored the fact that higher forgiveness implied temporal irregularity in the data, while all other approaches attempted to correct for the potential biases introduced by temporal irregularity. Note, the imputation approach is not affected by the forgiveness level, since it always starts with a full trajectory

Results were qualitatively similar for all three landscape autocorrelation scenarios and for different combinations of missingness and forgiveness (Appendix 1: Figure S4). Here, we report on results for a landscape with autocorrelation of 20, while either holding constant missingness at a conservative 20% (Fig. 5) or the forgiveness level at two (Fig. 6) (results for all other combinations are summarized in Additional file 1: Figure S4). The *imputation* approach resulted in biased estimators of $\beta _{dist}$ and $\beta _{forest}$, whereas all other approaches were able to recover the parameters of the habitat-selection function with minimal bias (Fig. 5). Note, the *imputation* approach always starts with a full trajectory and is therefore unaffected by the forgiveness level. For all other methods, increasing the forgiveness from 1 to 5 improved the precision of the estimators of habitat-selection parameters without introducing noticeable bias, with the biggest gains in precision and reduction in RMSE occurring when moving from a forgiveness of one to a forgiveness of two (Fig. 5). This highlights the potential benefits of leveraging additional data compared to the traditional approach, which uses only bursts of regular data (represented by the *uncorrected* approach and forgiveness = 1).

The *uncorrected*, *naïve*, and *imputation* approaches resulted in biased estimators of the parameters in the movement kernel, particularly for high values of forgiveness (Fig. 5a) and high levels of missingness (Fig. 6a). The *imputation* approach appeared to perform particularly poorly at estimating the concentration parameter of the turning-angle distribution (Fig. 6). The *multistep* and *dynamic+model* approaches resulted in unbiased estimators of parameters in the step-length distribution, but estimators of the concentration parameter exhibited a slight bias. This bias was, however, much smaller than we observed with the other approaches we considered. Increasing missingness negatively influenced the precision and accuracy of estimates, yet its impact could be dampened using the *dynamic+model* and *multistep* approaches (Fig. 6b).

## Case study

To showcase the applicability of the *dynamic+model* approach, which appeared to perform best with simulated data, we conducted a case study with real GPS data obtained on “Apollo”, a spotted hyena (*Crocuta crocuta*) inhabiting the Okavango Delta ecosystem in northern Botswana. Apollo’s data were collected between 2007 and 2011 using GPS radio collars (*GPS Plus*; Vectronic Aerospace GmbH, Berlin, Germany) and comprised 9316 GPS locations (details in [12] and [14]). Because hyenas are nocturnal [15], GPS collars were set to record data at two-hourly intervals between 18:00 and 06:00 o’clock, and to only record a single location at noon. For simplicity, we only considered nightly bursts and removed all locations obtained at noon. Missingness in this dataset was low (< 10 %, [14]) and to better showcase the usefulness of the *dynamic+model* approach, we thinned the data by randomly removing 25% of the obtained locations. As spatial covariate layers, we used Water, DistanceToWater and Trees (Appendix 1: Figure S5). Water was a binary variable representing major rivers and areas inundated by floodwater, whereas DistanceToWater was a continuous variable indicating the distance (in meters) to the nearest pixel categorized as water. Trees was a continuous variable indicating the percent tree cover in each pixel. We resampled all layers to a common resolution of 250 m ✕ 250 m and merged them into a single raster-stack (Appendix 1: Figure S5). The derivation of each covariate layer is described in detail in [34]. We dynamically fitted step-length and turning-angle distributions to steps with step durations of 2, 4, and 6 h, respectively, assuming a gamma distribution for step lengths and a von Mises distribution for turning angles. Instead of resampling the observed track to different step durations when fitting dynamic distributions (like we did in the simulation study), we introduced a larger amount of steps with step durations longer than two hours by thinning the data again (by another 10%). The benefit of this approach was that steps with irregular step durations occurred more randomly and were not limited to the hours specified by the resampling algorithm. Finally, we used iSSFs with the *dynamic+model* approach to estimate the habitat-selection function and movement kernel of Apollo. For this, we considered three cases:

- *F1*: We assumed a forgiveness of one (i.e., only steps with a regular step duration of 2 h), which is akin to conducting a traditional iSSA.
- *F3-S*: We assumed a forgiveness of three (i.e., considered steps with step durations of up to three times the regular step duration) and included interactions between the step duration ($\Delta t$) and step descriptors (sl, log(sl), and cos(ta)) in the regression model.
- *F3-SH*: We assumed a forgiveness of three (i.e., considered steps with step durations of up to three times the regular step duration) and included interactions between the step duration ($\Delta t$) and step descriptors (sl, log(sl), and cos(ta)), as well as between the step duration and habitat covariates in the regression model.

Notably, we included F3-SH to investigate if including interactions between the step duration and habitat-covariates would provide insights into scale-dependent habitat selection. In all cases, we generated 200 random steps and extracted spatial covariates at the end of observed and random steps. We then fit the three models using the conditional logistic regression framework as implemented in the survival R-package [69]. Lastly, we computed updated movement parameters for a regular step duration of 2 h.

**Fig. 7:**
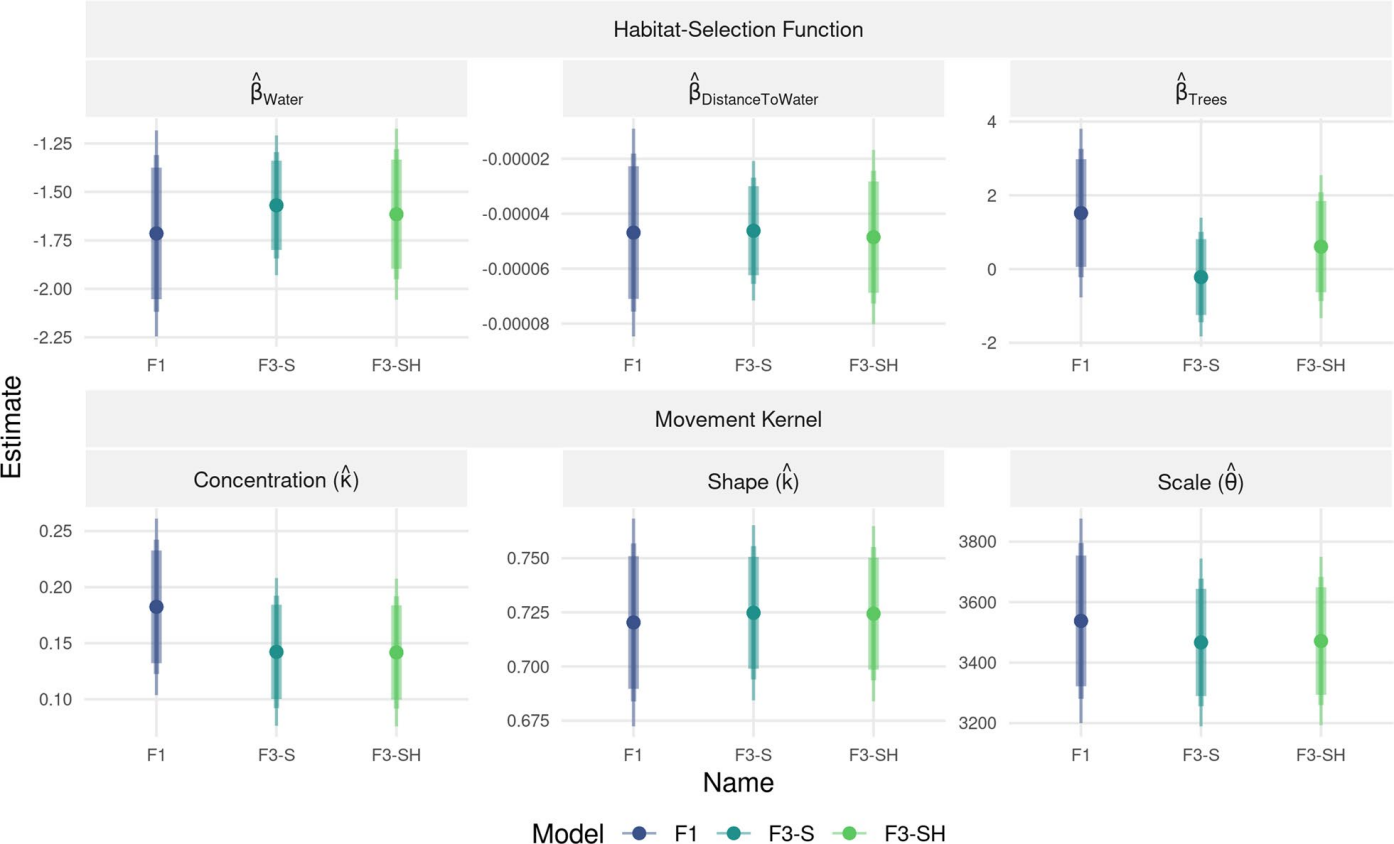
Model results from the case study using GPS data collected on Apollo. In F1, forgiveness was set to one (only 2-hour steps were considered), whereas in F3-S and F3-SH a forgiveness of three was employed (allowing for step durations of up to 6 h). In model F3-S, the step duration was interacted with step descriptors. In model F3-SH, step duration was interacted with step descriptors and habitat covariates. The bars indicate the 90%, 95%, and 99% confidence intervals. Note that for simplicity, we omitted interactions with the step duration from this figure

Results from the iSSF models show that increasing the level of forgiveness led to improvements in estimator precision (Fig. 7). This was achieved by increasing the effective sample size from 2179 to 4505 valid steps (Appendix 1: Table S1). The improvement in estimator precision was weaker for F3-SH than for F3-S, as the F3-SH model was more complex due to inclusion of additional interaction terms. Point estimates for the habitat-selection and movement parameters were similar for all 3 models, and evidence for scale dependency in habitat selection was fairly weak. F3-S and F3-SH had similar AIC scores ($\Delta AIC \le 1$; Appendix 1: Table S1), and the interaction terms were statistically significant only for the step duration of 6 h and only for one of the habitat covariates (Appendix 1: Table S1).

## Discussion

We conducted a simulation study with known habitat and movement parameters to investigate if retaining irregular animal locations via increased forgiveness improves or worsens parameter estimation in iSSFs. We also tested the performance of four different approaches that attempt to correct for potential biases introduced by using temporally irregular data, and we compared them to an uncorrected baseline approach. Our results demonstrated that retaining irregular animal locations can improve the precision of estimators of habitat-selection parameters but may lead to biased estimators of the parameters in the movement kernel. Overall, our results highlight the potential benefits of leveraging irregular animal locations, especially if an appropriate method for handling irregular data is chosen.

The *uncorrected* baseline approach ignored the fact that increasing forgiveness introduced irregularity in the data. Consequently, estimators of the parameters in the movement kernel were increasingly biased as forgiveness increased due to the inclusion of steps with varying step durations when fitting the model. Steps with longer step durations tended to have larger step lengths and less directed turning angles, which led to an overestimation of $\theta$ and underestimation of *k* and $\kappa$. Yet, estimators of the habitat-selection parameters remained unbiased or nearly so, even at high levels of forgiveness, and they were more precise than the standard estimator (represented by the *uncorrected* approach with forgiveness = 1). These results highlight the potential benefits that can be reaped by including additional data.

Similarly, the *naïve* approach performed well when estimating habitat-selection parameters but resulted in biased estimators of movement parameters, especially for large forgiveness values. This result was unsurprising given that our simulated trajectories were tortuous, and therefore, step lengths were not linearly related to step durations. Indeed, we found that, although there was a near linear relationship between step duration and the (tentative) scale parameter ($\theta _0$), the relationships between step duration and the (tentative) movement parameters $\kappa _0$ and $k_0$ were non-linear (Appendix 1: Figure S2). Overall, the usefulness of this approach appears highly limited, as it is often not clear by what factor distributional parameters for step lengths and turning angles should be multiplied to match the observed step duration.

The *dynamic+model* approach provided a flexible, easily implementable, and powerful framework for retrieving precise and unbiased estimators of the step-length and habitat-selection parameters, irrespective of the forgiveness level. The estimator of the concentration parameter exhibited some bias, but less than when using the *uncorrected* and *naïve* approaches. To implement the *dynamic+model* approach, we included interactions between step descriptors (sl, log(sl), and cos(ta)) and step duration in the conditional logistic regression model. This allowed the parameters of the movement kernel to depend on the step duration. A complication, however, is that turning angles are influenced by the step duration of both the current and previous step (Appendix 1: Figure S3). The bias in the concentration parameter likely arose from only accounting for the step duration associated with the current step and not the previous one. Moreover, fitting tentative distributions for different step durations can be challenging due to some step durations occurring only rarely. However, by resampling observed animal locations to different step durations using the track_resample function from the amt R-package [67] the needed data can easily be generated. We included step duration as a categorical covariate, yet there may be times when it would be advantageous to treat it as a continuous covariate (e.g., with its effect modeled using a low-degree polynomial or regression spline with few degrees of freedom). Treating step duration as a continuous variable may help to alleviate convergence issues in cases where some step durations are rare, and it might allow applying the *dynamic+model* approach to data that are entirely irregular.

The *multistep* approach also performed well and was relatively easy to implement. This approach is somewhat *ad hoc* in that it uses the tentative movement parameters to generate random steps to match observed steps with longer step durations (in multiples of $\Delta t$). It is similar to, but slightly less principled, than the approach developed by Vales [73], which formally constructs the likelihood for multistep durations by integrating out the missing steps. An advantage of this latter approach is that one can also attempt to account for non-random missingness by explicitly modeling factors related to the probability of obtaining a successful location [73]. Nonetheless, integrating over the missing steps, as in Vales [73], can be computationally intensive and prohibitive with large data sets. Another downside of both of these approaches (the *multistep* approach and the approach of [73]) is that they can only be applied in cases where step durations are a fixed multiple of the regular step duration; i.e., unlike the *dynamic+model* approach, they cannot be applied when data are highly irregular.

Of the methods we considered, the *imputation* approach performed the worst. It resulted in biased estimators of parameters in both the habitat-selection function and the movement kernel. This bias likely resulted from using an overly simplistic movement model to impute missing fixes. Moreover, the imputation procedure may have led to imputed animal locations that masked important selection properties, therefore leading to inaccurate parameter estimates. While this approach appears to perform well with hidden Markov movement models [50], we advise against its use with iSSFs.

For the scenarios we considered in our simulation study, the estimators of habitat-selection parameters were insensitive to the inclusion of irregular data and performed well, except for the *imputation* approach. This suggests that accounting for irregular step durations may not be particularly important if one is only interested in the habitat-selection function. When the movement kernel is also of interest, we suggest the *dynamic+model* approach, since it is flexible, easy to implement, and allows one to use more data than the traditional approach that requires bursts of regular data, leading to more precise estimators.

Several authors have emphasized that movement and habitat-selection parameters in an SSF are scale dependent and should be expected to change as the sampling frequency changes [see for example 3, 66, 27]. Furthermore, Barnett [4] developed an analytical framework for investigating scale dependence and showed that habitat-selection parameters should depend on the relative width of the movement kernel in relation to habitat heterogeneity. Thus, the relative insensitivity of the habitat-selection parameters to the inclusion of steps with varying step duration was somewhat unexpected. It would be interesting to explore the robustness of this result across a wider range of simulation scenarios in the future.

More generally, the spatial scale of a habitat-selection analysis has been recognized as an important factor, which is why Johnson [39] proposed a hierarchical framework for examining habitat-selection across different orders (e.g., species range, individual home range, within a home range). Johnson’s proposed framework acknowledges that habitat-selection may act differently at different scales, and that the interpretation of ecological processes changes depending on the spatial scale at which they are investigated [47, 75]. This understanding has encouraged scientists to conduct extensive scaling analyses and to comprehensively examine habitat-selection at multiple scales [17, 51, 59, 79]. In studies employing SSAs, the issue of scale is often neglected, and data are most frequently analyzed at the spatio-temporal scale at which they were collected. This choice maximizes the number of locations that can be used in the analysis, yet prevents a thorough understanding of scale dependency. The use of irregular data in SSAs poses another challenge, as steps with unequal step durations may reflect selection processes occurring at different scales. The severity of this issue obviously depends on the original sampling frequency, the degree of missingness, and the scale at which animals are making decisions that are relevant in terms of their movement behavior and habitat selection. By including irregular animal locations via increased forgiveness, we may therefore average over selection processes occurring at multiple scales, which could produce estimates of habitat-selection parameters that are misleading due to contradictory effect signs at different scales. To better account for such scale-dependent processes, it may be beneficial to include interactions between step duration and habitat features (e.g., dist, elev, forest), thus allowing habitat-selection parameters to also vary as a function of step duration. We demonstrated how this could be implemented in the case study.

It is important to note that we considered a limited number of scenarios in our simulation study. For instance, we assumed that animal locations were missing at random, i.e., failure to obtain a fix was unrelated to habitat types, time of the day, etc. However, several studies have shown that missingness is often non-random and related to difficulties with satellite transmission due to topography [48], canopy cover [18, 31, 58], time of the day [30], animal behavior [49], or collar orientation [20]. In fact, Vales [73] highlighted that missingness and the associated under-representation of certain habitat types may lead to biased estimators of parameters in iSSFs, but that accounting for the probability of obtaining a location in differing environmental conditions may alleviate this bias. Future studies should strive to further investigate these relationships and examine how our proposed approaches perform when missingness is habitat-dependent.

A major benefit of using iSSFs is the ability to allow an individual’s movement kernel to depend on local habitat features [3]. In our simulation study, we considered simplified scenarios where the movement kernel was unchanging, which simplified the simulation and inference. Nevertheless, such interactions often play a crucial role in real ecosystems. For instance, Dickie [21] employed iSSFs and revealed that several large mammal species moved faster while on linear features. Similarly, Hofmann [36] found that African wild dogs moved significantly slower and less directed in areas that were covered by floodwater. Future studies could investigate simulation scenarios in which individuals alter their movement tendencies in response to local environmental features (i.e., models with habitat dependent movement kernels) and examine how this influences the robustness of our proposed approaches.

While our results suggest that irregularity due to missing animal locations can effectively be accounted for in iSSFs and that increasing the forgiveness, thus allowing for inclusion of irregular data, improves estimator precision, we also found a decreasing marginal benefit of increased forgiveness. In fact, increasing the forgiveness beyond a value of two (i.e., allowing for steps of twice the regular step duration) only marginally improved model performance in our case. This can also be seen in Fig. 2, which shows that the largest number of steps that can be gained is when increasing the forgiveness level from one to two. Having a higher forgiveness beyond two may thus not even be necessary, therefore limiting the need to correct biases emerging from the inclusion of irregular data.

Although we focused on the case of missing location data, the proposed approaches may also prove useful for situations where sampling is irregular for other reasons. For example, it is not uncommon to adjust sampling regimes after a preliminary phase, following improvements to collar-battery-lifetime, or for sampling rates to vary depending on type and manufacturer of the collar [9]. Similarly, it is common practice to adjust the GPS regime to the biology of the focal species and only record data during a specific time of the day (e.g., [8, 12, 24]). These irregularities might be addressed using the *dynamic+model* approach, with interactions between step duration and movement descriptors. Interactions between step duration and habitat-selection covariates should also be considered, particularly if the sampling regime is adjusted to coincide with changes in animal behavior. We expect this approach will work fairly well in many cases, but we might expect a slight bias in the estimated concentration parameters, as observed in our simulation study.

Our study contributes to the growing body of literature that extends iSSFs and improves the method’s robustness under various conditions. This includes approaches for modeling irregular data [23, 55], accounting for spatial dependence among residuals [2], methodological frameworks for fitting iSSFs with random slopes [54] and random smooths [45], incorporating the probability of successfully obtaining an animal location in different habitat conditions [73], and considering the behavioral states of the tracked animals [44, 60].

In conclusion, our study shows that inclusion of irregular animal locations can improve model performance, yet only when an appropriate approach to account for irregularity is selected. Here, the *dynamic+model* and *multistep* approaches performed well and resulted in improved estimators of habitat-selection and movement parameters, even at elevated levels of missingness and forgiveness. Both methods are easy to implement, and the associated models can readily be fitted using the R-packages amt [67], survival [69], coxme [68], and mgcv [45, 77, 78]. To facilitate uptake and encourage use of the proposed approaches among practitioners, we provide all of our codes through an online repository, which includes an example application of the *dynamic+model* approach. With this, we hope practitioners will rethink the common use of discarding large portions of data and instead use methods that can accommodate irregular data.

### Supplementary Information

**Appendix**: Additional figures and tables.

## Data Availability

Code to reproduce this study is available through the University of Minnesota’s Data repository (https://doi.org/10.13020/6wcd-6s43, [35]). The repository also includes two example analyses that showcase the application of the *dynamic+model* approach to simulated data, as well as to the GPS data of Apollo.
