# Supplementary material for: Methods for implementing integrated step-selection functions with incomplete data
Source: Mov Ecol. 2024 May 9;12:37. doi: 10.1186/s40462-024-00476-8 (PMC11081933; doi:10.1186/s40462-024-00476-8)
Supplement: Supplementary file 1 — Appendix: Additional figures and tables. [file 40462_2024_476_MOESM1_ESM.pdf]

Appendix

Methods for Implementing Integrated Step-Selection Functions with Incomplete Data

David D. Hofmann[1,2,§] Gabriele Cozzi[1,2] John Fieberg [3]

February 14, 2024

[1] Department of Evolutionary Biology and Environmental Studies, University of Zurich, Winterthurerstrasse 190, 8057 Zurich, Switzerland.

[2] Botswana Predator Conservation Program, Wild Entrust, Private Bag 13, Maun, Botswana.

[3] Department of Fisheries, Wildlife, and Conservation Biology, University of Minnesota, St. Paul, MN, USA.

[§] Corresponding author: david.hofmann2@uzh.ch

Running Title: Step-Selection Analyses with Missing Data

Keywords: animal movement, gps data, imputation, incomplete data, missing fixes, step-selection analyses, step-selection functions

A.1 Landscape Simulation: Different Autocorrelation Scenarios

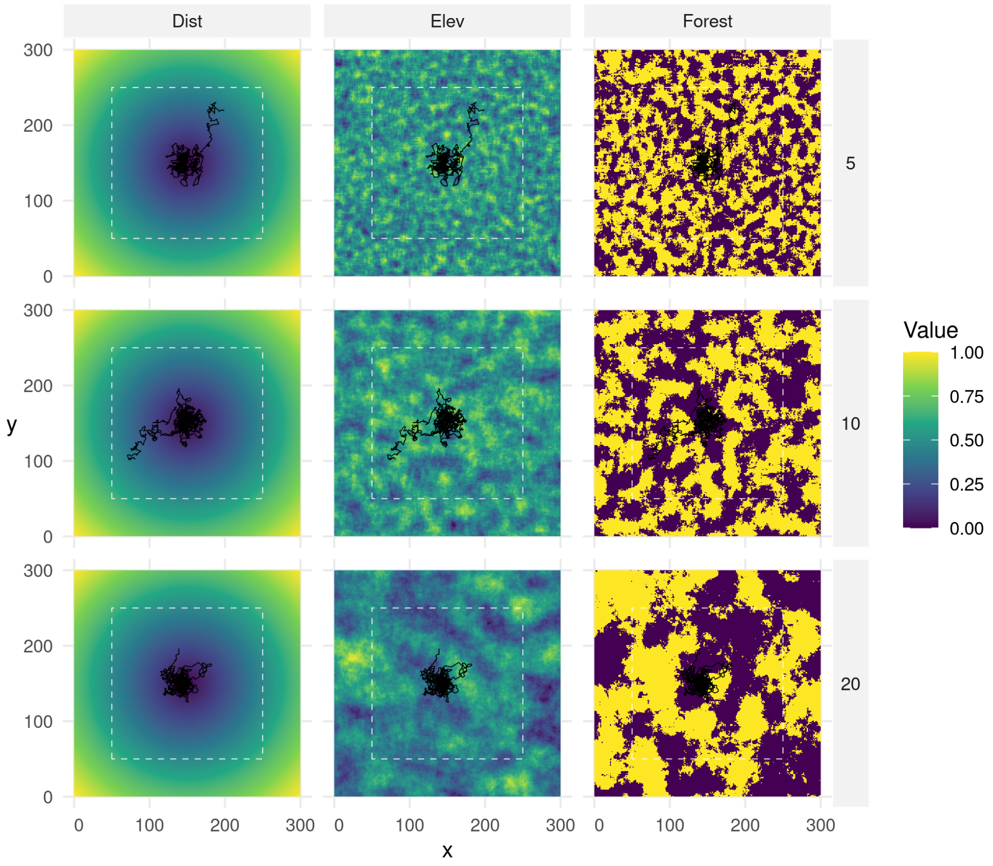

Figure S1: Simulated landscapes under different levels of autocorrelation (5, 10, 20; from top to bottom). Autocorrelation only affected the layers elev and forest, which were both simulated using a Gaussian random field neutral landscape model (Schlather et al., 2015) using the R-package NLMR (Sciaini et al., 2018). Simulations were repeated 100 times for each autocorrelation scenario, thus resulting in 300 unique landscape configurations.

A.2 Dynamic Tentative Distribution Parameters

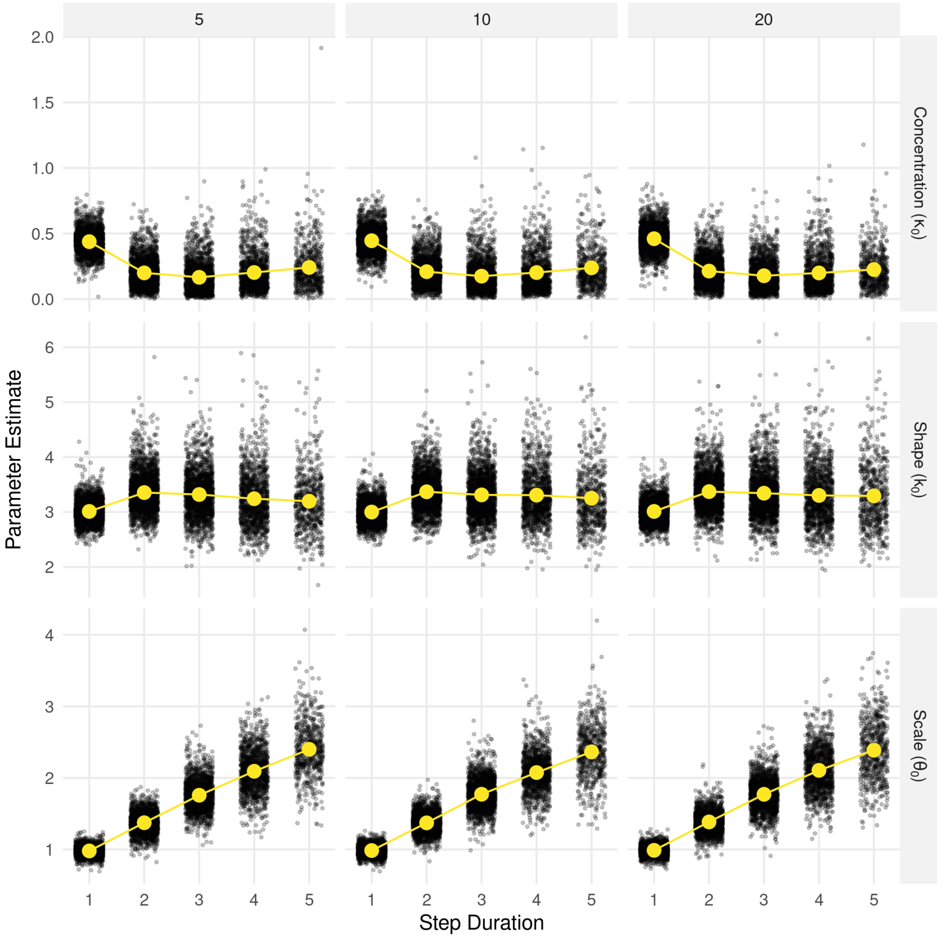

Figure S2: Tentative prameter estimates for the von Mises distribution (top row) and gamma distribution (bottom row) fitted to steps with different durations. The von Mises distribution requires one parameter, namely a concentration parameter (κ). The gamma distribution requires a shape parameter (k) and a scale parameter (θ). The subscript $_0$ is used to indicate that these are tentative distribution parameters (sensu Avgar et al., 2016 and Fieberg et al., 2021).

A.3 Distribution of Relative Turning Angles following Different Step Durations

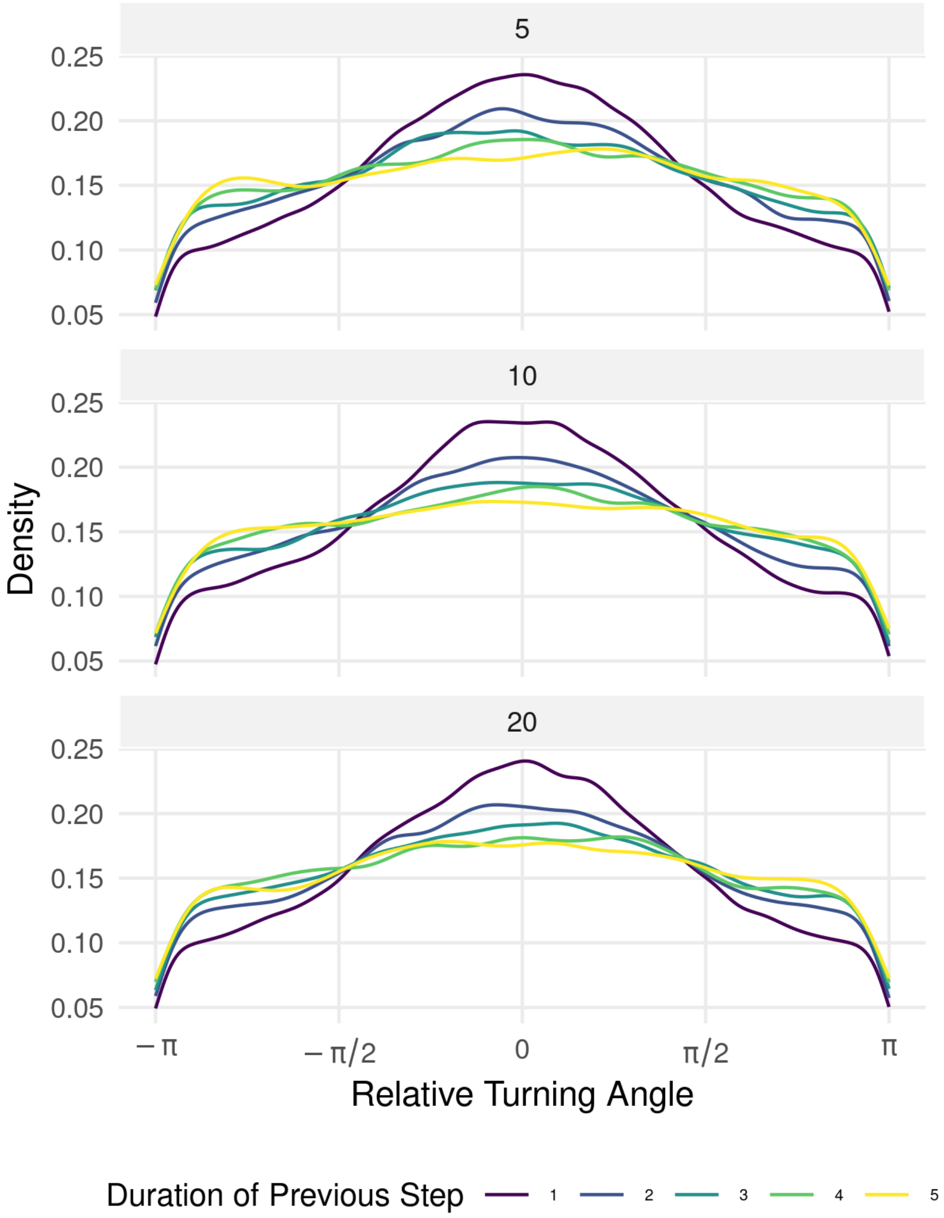

Figure S3: Density of relative turning angles associated with steps of $\Delta t = 1$ following steps with different durations for all three autocorrelation scenarios (5, 10, and 20). To generate this figure, we assumed a missingness of 0.5 and forgiveness of 5.

A.4 Model Estimates across all Scenarios

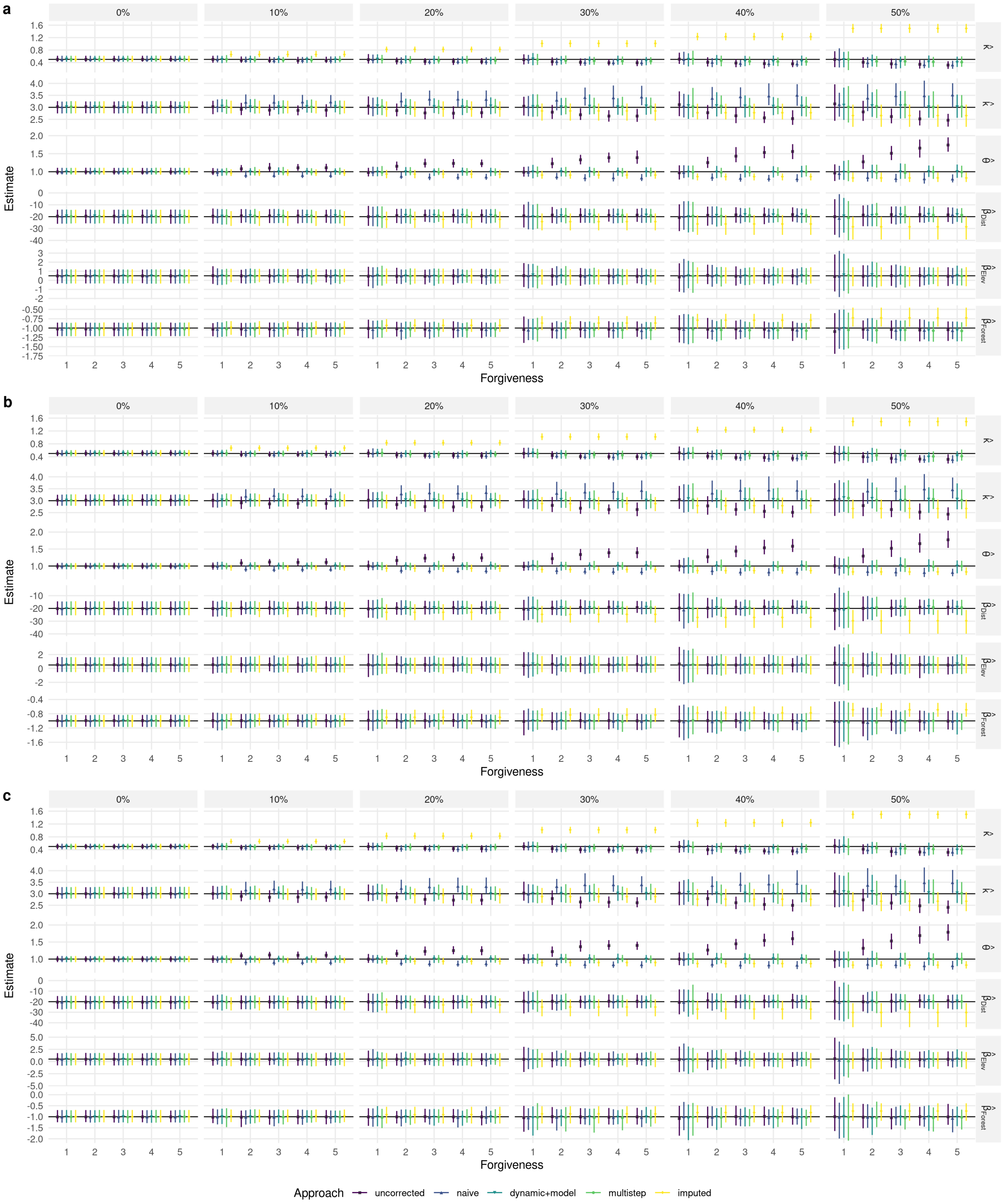

Figure S4: Parameter estimates across different autocorrelation scenarios (5, 10, 20; panels a, b, and c) and missingness levels (0% - 50%; from left to right). True simulation parameters are indicated by the solid black lines. Parameter estimates from the different approaches are given by the colored symbols, and their bootstrap 95% CIs across 100 replicates by the colored lines.

A.5 Case Study Covariates

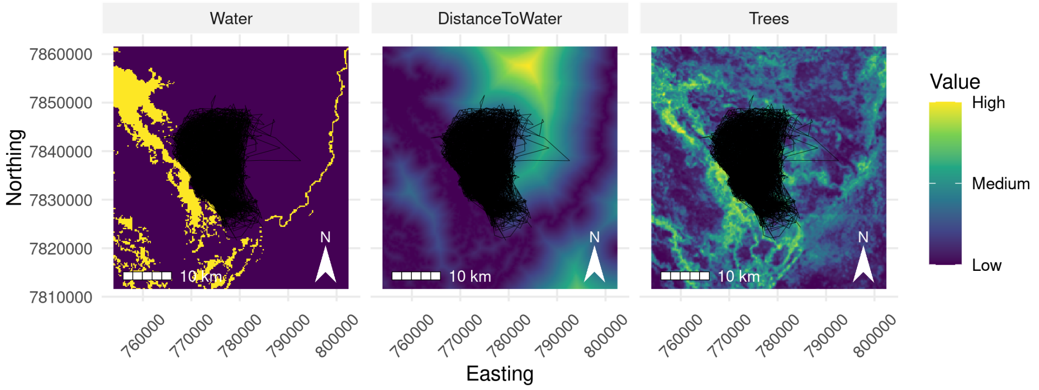

Figure S5: Covariates used for the case study, overlaid with the GPS data of a spotted hyena called "Apollo" (lines in black). Apollo was originally collared in 2007 in northern Botswana and monitored until 2011. The depicted area is part of the Okavango Delta, which is a massive wetland area. Data was projected to a local projection (EPSG:32734).

A.6 Case Study Model Output

Table S1: Model results from the case study using GPS data collected on Apollo. In F1, forgiveness was set to one (only 2-hour steps were considered), whereas in F3-S and F3-SH a forgiveness of three was employed (allowing for step durations of up to 6 hours). In model F3-S, the step duration was interacted with step descriptors. In model F3-SH, step duration was interacted with step descriptors and habitat covariates.

Coefficient	F1	F3-S	F3-SH
sl	0.00002	0.00001	0.00001
	(0.00001)	*(0.00001)*	*(0.00001)*
log_sl	-0.02251	-0.01805	-0.01846
	(0.01862)	*(0.0157)*	*(0.0157)*
cos_ta	0.03166	-0.0085	-0.009
	(0.03055)	*(0.02557)*	*(0.02558)*
Water	-1.71418***	-1.56915***	-1.61501***
	(0.20628)	*(0.13995)*	*(0.17121)*
DistanceToWater	-0.00005***	-0.00005***	-0.00005***
	(0.00001)	*(0.00001)*	*(0.00001)*
Trees	1.51764*	-0.2178	0.60764
	(0.88816)	*(0.62517)*	*(0.75259)*
sl:duration4		-0.00001	-0.00001
		(0.00002)	*(0.00002)*
sl:duration6		-0.00004**	-0.00004**
		(0.00002)	*(0.00002)*
log_sl:duration4		0.08122	0.07978
		(0.05075)	*(0.0508)*
log_sl:duration6		0.02867	0.03058
		(0.02471)	*(0.02474)*
cos_ta:duration4		-0.07526	-0.07635
		(0.05784)	*(0.05788)*
cos_ta:duration6		-0.16358***	-0.16105***
		(0.06055)	*(0.06059)*
Water:duration4			0.08548
			(0.34111)
Water:duration6			0.20782
			(0.46194)
DistanceToWater:duration4			0.00002
			(0.00002)
DistanceToWater:duration6			-0.00001
			(0.00003)
Trees:duration4			-0.04696
			(1.61148)
Trees:duration6			-6.73474***
			(1.97442)
Steps	2,179	4,505	4,505
AIC	-	47,565	47,564

Significance codes: * $p < 0.10$ ** $p < 0.05$ *** $p < 0.01$

References

Avgar, T., Potts, J. R., Lewis, M. A., & Boyce, M. S. (2016). Integrated Step Selection Analysis: Bridging the Gap Between Resource Selection and Animal Movement. *Methods in Ecology and Evolution*, *7*(5), 619–630. https://doi.org/10.1111/2041-210X.12528

Fieberg, J., Signer, J., Smith, B., & Avgar, T. (2021). A 'How to' Guide for Interpreting Parameters in Habitat-Selection Analyses. *Journal of Animal Ecology*, *90*(5), 1027–1043. https://doi.org/10.1111/1365-2656.13441

Schlather, M., Malinowski, A., Menck, P. J., Oesting, M., & Strokorb, K. (2015). Analysis, Simulation and Prediction of Multivariate Random Fields with Package RandomFields. *Journal of Statistical Software*, *63*, 1–25. https://doi.org/10.18637/jss.v063.i08

Sciaini, M., Fritsch, M., Scherer, C., & Simpkins, C. E. (2018). Nlmr and Landscapetools: An Integrated Environment for Simulating and Modifying Neutral Landscape Models in R. *Methods in Ecology and Evolution*, *9*(11), 2240–2248. https://doi.org/10.1111/2041-210X.13076
